# Pandemic preparedness: why humanities and social sciences matter

**DOI:** 10.3389/fpubh.2024.1394569

**Published:** 2024-08-16

**Authors:** Sally Frampton, Kingsley Orievulu, Philippa C. Matthews, Alberto Giubilini, Joshua Hordern, Lizzie Burns, Sean Elias, Ethan Friederich, Nomathamsanqa Majozi, Sam Martin, Austin Stevenson, Samantha Vanderslott, Janet Seeley

**Affiliations:** ^1^Faculty of History, University of Oxford, Oxford, United Kingdom; ^2^Africa Health Research Institute, Durban, South Africa; ^3^Centre for China-Africa Studies, University of Johannesburg, Johannesburg, South Africa; ^4^School of Nursing and Public Health, University of KwaZulu-Natal, Durban, South Africa; ^5^The Francis Crick Institute, London, United Kingdom; ^6^Division of Infection and Immunity, University College London, London, United Kingdom; ^7^Department of Infectious Diseases, University College London Hospital, London, United Kingdom; ^8^Nuffield Department of Medicine, University of Oxford, Oxford, United Kingdom; ^9^Uehiro Oxford Institute, Humanities Division, University of Oxford, Oxford, United Kingdom; ^10^Faculty of Theology of Religion, Harris Manchester College, University of Oxford, Oxford, United Kingdom; ^11^Department of Pharmacology, University of Oxford, Oxford, United Kingdom; ^12^Pandemic Sciences Institute, Nuffield Department of Medicine, University of Oxford, Oxford, United Kingdom; ^13^Wellcome Centre for Ethics and Humanities, Ethox Centre and Centre for the History of Science, Medicine and Technology, University of Oxford, Oxford, United Kingdom; ^14^Oxford Vaccine Group, University of Oxford and NIHR Oxford Biomedical Research Centre, Oxford, United Kingdom; ^15^Rapid Research Evaluation and Appraisal Lab (RREAL), Department of Targeted Intervention, University College London (UCL), London, United Kingdom; ^16^School of Ministry, Palm Beach Atlantic University, West Palm Beach, FL, United States; ^17^Department of Global Health and Development, London School of Hygiene and Tropical Medicine, London, United Kingdom

**Keywords:** pandemic preparedness, vaccination, vaccine hesitancy, humanities, history, ethics, religion, social sciences

## Abstract

Whilst many lessons were learned from the COVID-19 pandemic, ongoing reflection is needed to develop and maintain preparedness for future outbreaks. Within the field of infectious disease and public health there remain silos and hierarchies in interdisciplinary work, with the risk that humanities and social sciences remain on the epistemological peripheries. However, these disciplines offer insights, expertise and tools that contribute to understanding responses to disease and uptake of interventions for prevention and treatment. In this Perspective, using examples from our own cross-disciplinary research and engagement programme on vaccine hesitancy in South Africa and the United Kingdom (UK), we propose closer integration of expertise, research and methods from humanities and social sciences into pandemic preparedness.

## Introduction: interdisciplinary pandemic preparedness—an urgent need

In the aftermath of COVID-19, ‘pandemic preparedness’ became part of a global conversation (1). In September 2021, as the world grappled with the devastating consequences of the virus, the United States Government released a paper advocating for pandemic readiness akin to ‘the seriousness of purpose, commitment, and accountability of the Apollo Program’ (2). The effects of climate change are likely to aggravate the spread of pathogenic disease, requiring rigorous preparation through innovation and investment in infrastructure (3). A growing number of research institutions and multisectoral initiatives have emerged which focus on pandemic preparedness (4).

In December 2021, the World Health Assembly formed the Intergovernmental Negotiating Body to create a global accord on ‘pandemic prevention, preparedness and response’ (5). Three months later the World Health Organization (WHO) Advisory Group for Behavioural Insights and Sciences issued an open letter to the Negotiating Body, asking them to ‘explicitly include the behavioural and social sciences in the new international instrument as they are critical for pandemic prevention, preparedness and response’ (6). The Group’s plea demonstrates the difficulties still apparent for social sciences and humanities scholars in getting a seat at the table in discussions around pandemic preparedness and response. Global health policy expert Clare Wenham at the London School of Economics has criticised the proliferation of pandemic preparedness institutes, arguing they are often ‘the same people, considering the same issues’ (7). Wenham’s argument pertains primarily to the continued agenda setting by high-income, Global North countries. However, her argument can also be brought to bear on the relatively narrow disciplinary range contributing to pandemic preparedness. We contend that humanities and social sciences must be regarded as equal contributors, because preparedness and response are primarily about human behaviour, and because public health policies are influenced by cultural, moral and political values. Humanities and social sciences are crucial in understanding attitudes, beliefs, and behaviours relevant to all public health and policy measures, including acceptance and uptake of vaccines as well as other aspects of infection control.

In 2019—before the first case of SARS-coV-2 infection had been identified—we formed an interdisciplinary group to lead the ‘Infecting Minds’ programme, to merge historical, anthropological, ethical, theological, clinical, and science communication perspectives to explore vaccine acceptance and hesitancy. We sought to explore vaccine acceptance and hesitancy in two settings within the KwaZulu-Natal Province of South Africa, considering how attitudes towards vaccines change according to geographical locale and demographics (considering eThekwini, the Municipality which includes the city of Durban, and the predominantly rural area of uMkhanyakude District in the north of the Province). Alongside this, we established a public engagement programme that ran across Oxford (UK) and Durban and uMkhanyakude District (South Africa) to explore the perspective of 11-18 year olds towards vaccines.

We could not have known how prescient the project would be when in 2020 the COVID-19 pandemic emerged. Despite the interruptions of the pandemic, this work was completed in 2023 (8). In this piece, we briefly present the perspectives, opinions and insights that have emerged from the ‘Infecting Minds’ programme, and set out the benefits of investment in this shared approach to pandemic preparedness.

## Values and beliefs

Pandemic preparedness requires a continuous readiness to understand and engage with values and beliefs that shape competing viewpoints on public health. These will vary across different communities and different individuals, being informed by past contexts and current experiences, and potentially changing over time. Narratives might be centred on values such as the common good, individual freedom, religious obligations, national solidarity, and so on. Which values and beliefs we pursue, and how we navigate conflicts between them, are ethical-political choices, which, whilst perhaps informed by clinical or laboratory science, are not dictated by them. Decisions based on scientific evidence can be complicated by conflicts about values which underlie the moral, political and, in some contexts, religious complexities of phenomena like vaccine hesitancy or refusal.

Those who refuse vaccines can be labelled as simply scientifically ‘ill-informed’. But most of those who accept vaccines are also largely uninformed about the intricacies of vaccine science, and those who decline or question vaccines can be quite knowledgeable about them. Assuming a lack of understanding as the root cause of vaccine hesitancy fails to do justice to the complexity of human approaches to – and decision-making about – health and our bodies. Moreover such thinking can create barriers to working creatively and collaboratively with communities. When it comes to vaccine confidence and refusal to be vaccinated, we are often not as confounded by our science as by our humanity.

## A ‘tailored approach’ to pandemic preparedness: localities of hesitancy

The WHO has emphasised the need for a ‘tailored’ approach to pandemic preparedness. A complex nexus of drivers shapes the outlook and attitude of populations towards vaccines within a particular locale, impacting on vaccine confidence. In our study in KwaZulu-Natal, we conducted 30 in-depth interviews with citizens, traditional healers and healthcare practitioners, and found that vaccine confidence was shaped by fears about the effectiveness and safety of the COVID-19 vaccines relative to the timelines of their development (9). Social media, and communal engagement in the dissemination of ideas, were also important influencers. Suspicion of European and North American experimental research also emerged in some responses, understandably, given the post-apartheid context, and communities where Afrocentric (decolonised) ideas have gained stronger momentum.

Humanities can enhance the findings of such qualitative social science research. Our study, for example, invites further scrutiny from theology and history. Perceptions and attitudes towards vaccines were often borne out of people’s interpretations of religious teachings alongside their interactions with clinical medicine. Amongst most mainstream Christian denominations, congregations generally aligned with the vaccination drive – although the perspective of an ‘end time’ agenda, linked to vaccines as being associated with the ‘mark of the beast’, resonated amongst some Christian people in our study, corroborating other studies on the circulation of antivaccine theories amongst evangelical and charismatic denominations in South Africa (10). Some traditional healers explained how acceptance of vaccines was constrained by their ancestral beliefs: accepting external (and western) medicines could have serious spiritual ramifications. These narratives highlight the need to recognise the nuances of socio-theological influences on engagements with public health interventions.

Pandemic preparedness should incorporate such deeply human dimensions rather than marginalise them. The humanities and social sciences open opportunities to deepen an understanding of how cultural and social values and traditions shaping lives and belief systems could affect and perhaps even enhance vaccine acceptance. Various studies in other national contexts have provided evidence of the benefits of this socially and religiously literate approach (11–13).

## History matters

Historical perspectives can be useful when exploring the present and discussing the future of pandemic preparedness. The unique historical context of a country or region can inform vaccine hesitancy or acceptance. The methodologies used by historians, their approach to data and source material from the past, have been carefully honed to explore how and why events unfolded the way they did. An emergent theme during the COVID-19 pandemic was the seeming *lack* of preparedness in many parts of the world. Scholars have utilised historical perspectives to show how this came to be, exposing the impact of bureaucratic and political change, which led to an ‘atrophy of vigilance’ in some places, with direct consequences (14, 15).

Historical framings of both disease and their management are equally important (16–18). Within the history of infectious disease, it is common to present the long narrative of those on one side who championed medical progress and those on the other who defied it, particularly through resistance to vaccines. Whilst this perspective may build support for science, the reality is much more complicated. Even if misinformed or incorrect by present standards, those in the past had some rationale for vaccine refusal. Historically driven, policy-oriented programmes are not only feasible, but their benefits have already been evidenced by projects such as the WHO-affiliated Global Health Histories initiative (19).

Taking a single national context, South Africa, we can see the interplay between past events and present-day culture. Continuing health inequalities, uneven vaccine access and vaccine mistrust are challenges that have been seeded by the country’s history of exploitation by colonial forces (20). Historical events continue to show themselves in the evolving frameworks used to articulate vaccine inequity. The term ‘vaccine apartheid’, has been used in the last couple of years – including by the WHO Director-General—to describe the structurally embedded inequality to global vaccine access. The legacy of apartheid highlights the embeddedness of structural racism and colonial models of power within the vaccine ecosystem (21).

## Language, design and engagement with all

The importance of language, communication, and the presence of public engagement experts within the conversation cannot be underestimated. Twentieth-century discussions of modern vaccinology, mRNA vaccines and viral vectors were largely limited to the science-literate and actively engaged public. With time, biomedical scientific communication has improved, on account of a better understanding of the perils and pitfalls of hierarchical, top-down models of knowledge dissemination. However, the COVID-19 pandemic highlighted continued lacunae in understandings of vaccines, on the part of the public, the media, and politicians globally. For example, there were problematic misconceptions that the COVID-19 vaccine would ‘eradicate’ the virus, rather than focusing on its role to reduce the risk of infection and severe symptoms and/or long-term complications. Communication efforts were, at least initially, highly reactionary, due to a lack of capacity and time. This meant that careful presentation of topics such as defining what ‘efficacy’ means, vaccine safety in pregnancy, and the significance of rare side effects often came too late to prevent at least some damage to public confidence.

The pandemic highlighted acutely the role of social media in promoting and amplifying vaccine misinformation and driving hesitancy. There is progressive awareness that combating vaccine hesitancy and building vaccine confidence in the modern age requires a multitude of novel and targeted approaches to influence an increasingly diverse public opinion base. This involves a clear understanding of variations in the communication skills and media-related values of different generations. Our public engagement work with teenage populations in South Africa and the UK rasied the question of how communications might operate in the face of ambivalent views on vaccines and infectious disease, and tensions between parental or caregiver views and the views of young people (22, 23). Constructing a nuanced approach sensitive to generational difference requires innovative, interdisciplinary thinking. Creative tools from design and literature are essential in bridging health literacy in public health (24). What if those who could provide expertise on visual and textual communication, artists, graphic designers, writers, and content creators were invited into the conversation on pandemic preparedness from the beginning?

## Conclusion

Policy-relevant, interdisciplinary work is enormously challenging and requires sensitive collaboration as well as awareness of power dynamics between disciplines (25). It also requires political will; innovative approaches to pandemic preparedness will not flourish when the terms of engagement set by politicians exult the dominance of STEM approaches above all others. Developing, funding, and growing an integrated interdisciplinary approach is a global health priority which has the potential for innovating research agendas and enhancing cross-cultural preparedness for pandemics.

## Data Availability

The original contributions presented in the study are included in the article/supplementary material, further inquiries can be directed to the corresponding author.

## References

[1] Google Books NGram Viewer. Available online at: https://books.google.com/ngrams/graph?content=pandemic+preparedness&year_start=1800&year_end=2019&corpus=en-2019&smoothing=3 (n.d.). (Accessed 15 February 2024).

[2] American Pandemic Preparedness. Available online at: https://www.whitehouse.gov/wp-content/uploads/2021/09/American-Pandemic-Preparedness-Transforming-Our-Capabilities-Final-For-Web.pdf?page=29 (2021). (Accessed 15 February 2024).

[3] Mora C McKenzie T Gaw IM Dean JM von Hammerstein H Knudson TA. Over half of known human pathogenic diseases can be aggravated by climate change. Nat Clim Chang. (2022) 12:869–75. doi: 10.1038/s41558-022-01426-1, PMID: 35968032 PMC9362357

[4] The Rockefeller Foundation Pandemic Prevention Initiative. Institutions formed in the last few years include the Pandemic Institute in Liverpool, UK. Oxford, UK: The Pandemic Sciences Institute (2021).

[5] World Health Organization. Available online at: https://inb.who.int/ (2021). (Accessed 19 February 2024).

[6] WHO Technical advisory group on behavioral insights and sciences for health. Behavioural and social sciences are critical for pandemic prevention, preparedness and response. (2022). Available online at: https://www.who.int/news-room/commentaries/detail/behavioural-and-social-sciences-are-critical-for-pandemic-prevention-preparedness-and-response (Accessed 19 February 2024).

[7] Wenham C. Creating more and more new institutions may not make the world safer from pandemics. PLOS Glob Public Health. (2023) 3:e0001921. doi: 10.1371/journal.pgph.0001921, PMID: 37195920 PMC10191324

[8] University of Oxford. For resources relating to our schools engagement program, including a film of the events. (2023). Available online at: https://figshare.com/collections/_Infecting_Minds_Vaccine_Hesitancy_Project_Resources_Materials_and_Outputs/3498642/3 (Accessed 19 February 2024).

[9] Orievulu K S Frampton S Matthews P C Mpanza N Mjilo TF Nxumalo S. Infecting minds: perceptions and attitudes towards vaccines among rural and urban dwellers in the context of the COVID-19 pandemic in KwaZulu-Natal, South Africa. (2023), Preprint (Version 1) available at Research Square.

[10] Netshapapame TS. COVID-19 vaccination hesitancy in South Africa: biblical discourse. HTS Teol Stud. (2023) 79:a7795. doi: 10.4102/hts.v79i4.7795

[11] Nasiru SG Aliyu GG Gasasira A Aliyu MH Zubair M Mandawari SU. Breaking community barriers to polio vaccination in northern Nigeria: the impact of a grass roots mobilization campaign. Pathog Glob Health. (2012) 106:166–71. doi: 10.1179/2047773212Y.0000000018, PMID: 23265374 PMC4001576

[12] Oyo-Ita A Bosch-Capblanch X Ross A. Effects of engaging communities in decision-making and action through traditional and religious leaders on vaccination coverage in Cross River state, Nigeria: a cluster-randomised trial. PLoS One. (2021) 16:e0248236–6. doi: 10.1371/journal.pone.0248236, PMID: 33861742 PMC8051768

[13] Hall V Banerjee E Kenyon C Strain A Griffith J Como-Sabetti K. Measles outbreak—Minnesota April–may 2017. MMWR Morb Mortal Wkly Rep. (2017) 66:713–7. doi: 10.15585/mmwr.mm6627a1, PMID: 28704350 PMC5687591

[14] Witek TJ Schwartz R. The evolution of vigilance and its atrophy preceding the COVID-19 global pandemic. Front Public Health. (2022) 10:789527–7. doi: 10.3389/fpubh.2022.789527, PMID: 35664126 PMC9157789

[15] Busenberg G J. Policy Lessons from the History of Pandemic Preparedness *COVID-19 Rapid Response Impact Initiative* White Paper (2020) Available online at: https://ethics.harvard.edu/sites/hwpi.harvard.edu/files/center-for-ethics/files/23pandemicpreparedness.pdf?m=1600110919 (Accessed 25 February 2024).

[16] Nair A. Vaccinating against Vasoori: eradicating smallpox in the ‘model’ princely state of Travancore, 1804–1946. Indian Econ Soc Hist Rev. (2019) 56:361–86. doi: 10.1177/0019464619873798

[17] Flint K. “Africa Isn’t a testing lab”: considering COVID vaccine trials in a history of biomedical experimentation and abuse. J West Afric History. (2020) 6:126–40. doi: 10.14321/jwestafrihist.6.2.0126

[18] Milward G. Vaccinating Britain: Mass Vaccination and the Public Since the Second World War. Manchester: Manchester University Press (2019). 31487128

[19] Bhattacharya S Medcalf A Ahmed A. Humanities, criticality, and transparency: global health histories and the foundations of inter-sectoral partnerships for the democratisation of knowledge. Human Soc Sci Commun. (2020) 7:1–11. doi: 10.1057/s41599-020-0491-7 PMC7116415 33241231

[20] Thabethe S Slack C Lindegger G. “Why Don’t you go into suburbs? Why are you targeting us?”: trust and mistrust in HIV vaccine trials in South Africa. J Empir Res Hum Res Ethics. (2018) 13:525–36. doi: 10.1177/1556264618804740, PMID: 30417754 PMC6238163

[21] Sirleaf M. We charge vaccine apartheid? J Law Med Ethics. (2022) 50:726–37. doi: 10.1017/jme.2023.14, PMID: 36883405

[22] Burns E Ingamells T Frampton S Matthews P C. Polarised views or ambivalence? Vaccines through teenage eyes. PLOS Blogs (2023) Available online at: https://yoursay.plos.org/2023/01/polarised-views-or-ambivalence-vaccines-through-teenage-eyes/ (Accessed 25 February 2024)

[23] Infecting Minds. ‘Infecting Minds film’ (2023) Available online at: https://infectingminds.web.ox.ac.uk/video (Accessed 25 February 2024).

[24] Crawford P. Introduction: global health humanities and the rise of creative public health In: Crawford P Brown G Charise E, editors. The Routledge Companion to Health Humanities. London: Routledge (2020). 1–7.

[25] Frickel S Mathieu A Prainsack B. Introduction: investigating Interdisciplinarities In: Frickel A Prainsack H, editors. Investigating interdisciplinary collaboration: Theory and practice across disciplines. New Brunswick: Rutgers University Press (2017). 5–24.

